# The Environment as an Unrecognized Reservoir for Community-Associated Methicillin Resistant *Staphylococcus aureus* USA300: A Case-Control Study

**DOI:** 10.1371/journal.pone.0022407

**Published:** 2011-07-26

**Authors:** Anne-Catrin Uhlemann, Justin Knox, Maureen Miller, Cory Hafer, Glenny Vasquez, Megan Ryan, Peter Vavagiakis, Qiuhu Shi, Franklin D. Lowy

**Affiliations:** 1 Division of Infectious Diseases, Department of Medicine, College of Physicians and Surgeons, Columbia University, New York, New York, United States of America; 2 Mailman School of Public Health, Columbia University, New York, New York, United States of America; 3 Panna Technologies, Inc., New York, New York, United States of America; 4 Department of Epidemiology and Biostatistics, School of Public Health, New York Medical College, Valhalla, New York, United States of America; 5 Department of Pathology, College of Physicians and Surgeons, Columbia University, New York, New York, United States of America; National Institutes of Health, United States of America

## Abstract

**Background:**

Community-associated methicillin-resistant *Staphylococcus aureus* (CA-MRSA) infections are spreading, but the source of infections in non-epidemic settings remains poorly defined.

**Methods:**

We carried out a community-based, case-control study investigating socio-demographic risk factors and infectious reservoirs associated with MRSA infections. Case patients presented with CA-MRSA infections to a New York hospital. Age-matched controls without infections were randomly selected from the hospital's Dental Clinic patient population. During a home visit, case and control subjects completed a questionnaire, nasal swabs were collected from index respondents and household members and standardized environmental surfaces were swabbed. Genotyping was performed on *S. aureus* isolates.

**Results:**

We enrolled 95 case and 95 control subjects. Cases more frequently reported diabetes mellitus and a higher number of skin infections among household members. Among case households, 53 (56%) were environmentally contaminated with *S. aureus*, compared to 36 (38%) control households (p = .02). MRSA was detected on fomites in 30 (32%) case households and 5 (5%; p<.001) control households. More case patients, 20 (21%) were nasally colonized with MRSA than were control indexes, 2 (2%; p<.001). In a subgroup analysis, the clinical isolate (predominantly USA300), was more commonly detected on environmental surfaces in case households with recurrent MRSA infections (16/36, 44%) than those without (14/58, 24%, p = .04).

**Conclusions:**

The higher frequency of environmental contamination of case households with *S. aureus* in general and MRSA in particular implicates this as a potential reservoir for recolonization and increased risk of infection. Environmental colonization may contribute to the community spread of epidemic strains such as USA300.

## Introduction

The dramatic increase in skin and soft tissue infections caused by CA-MRSA remains a major public health issue [1], [2], often afflicting young, healthy individuals [3], [4]. These strains have now become endemic in many communities worldwide [1], [5]. Remarkably, only a few clones, including USA300 in the United States [6], have driven this epidemic [7]. The successful spread of CA-MRSA strains suggests unique features that facilitate their transmission and persistence.

Outside of epidemic CA-MRSA outbreaks, the major burden of *S. aureus* acquisition, transmission and disease appears to be contained within community households [8], [9], [10]. Frequently, CA-MRSA infections recur in affected individuals or are dispersed among members of the same household [8], [9], [11]. However, the source of these infections remains unclear. For example, nasal colonization appears to be less of a risk factor for infections [12], [13], [14], which is in contrast to hospital-acquired strains, where nasal MRSA carriage has clearly been established as a risk factor for subsequent infections with the same strain [15], [16]. This would suggest a role for other body sites [17] or perhaps the environment [14] as reservoirs for acquisition and subsequent *S. aureus* infections.

Anecdotal evidence provides support for the role of the environment as a source of CA-MRSA infections. In case reports, individuals who received several months of appropriate antibiotic treatment were cured only after successful decontamination of colonized household surfaces [18], [19]. In addition, MSSA and MRSA have been recovered from multiple areas in homes without apparent infections [20], [21].

To inform the innovative strategies that are clearly required to disrupt the ongoing CA-MRSA epidemic, it is critical to define the sources and reservoirs of *S. aureus* transmission and infections. We conducted a case-control study to examine the role of socio-demographic risk factors and environmental household contamination in patients with CA-MRSA infections compared with healthy controls living in the same community.

## Methods

### Ethics

Written informed consent was obtained from each individual before conducting an interview or obtaining samples. We received parental consent for participating children <18 years old, and pediatric assent was obtained from those capable of providing it. Index participants were compensated $10 for their time. The Institutional Review Board of Columbia University Medical Center, New York, United States approved this study.

### Study population

**Figure 1:**
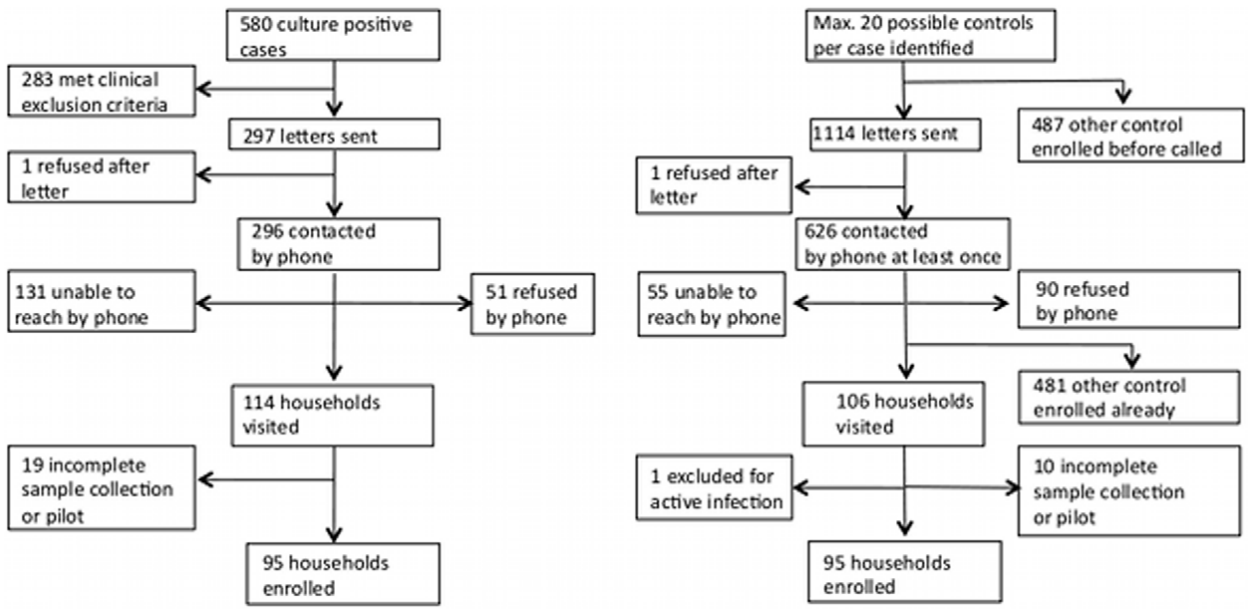
Flow chart enrollment of cases and controls.

This study took place between January 2009 and May 2010 as part of an ongoing case-control study on transmission of CA-MRSA in the Northern Manhattan community, defined by zip codes adjacent to the Columbia University Medical Center (CUMC). We identified 580 patients with positive MRSA cultures from wound, blood, urine or sputum specimens, obtained from outpatients or inpatients within 72h of admission (**Figure 1**). Patients were ineligible if they were either: a resident in a long-term care facility or hospitalized within the past 6 months, homeless or living in a shelter, having a chronic illness such as dialysis, or younger than 2 years. Upon review of their medical records, 297 patients met the study inclusion criteria. We contacted 296 patients by telephone; 131 could not be reached, 52 refused and 114 (38.5%) persons agreed to participate in the study and were enrolled.

Potential control participants were randomly selected from a database listing all patients attending the CUMC dental clinic. Cases and controls were matched by age (±2 years) and date of positive culture to date of dental clinic visit (±2 weeks). Up to 20 possible controls (range 3–20) per case were contacted by mail simultaneously (1114 total). Controls underwent the same enrollment procedures as cases (**Figure 1**). Overall, 626 possible controls were called by telephone at least once, one by one, until one agreed to participate, and 106 matched controls were identified and visited at home. Case and matching control interview were, on average, carried out within 30 days of each other (range 7–91 days).

Nineteen case participants and eleven control respondents were subsequently excluded because they were part of the piloting process of the study, or were not interviewed at their residence, thus precluding assessment of their home environmental contamination. We therefore included 95 patients with CA-MRSA infections and 95 age-matched controls without evidence of current *S. aureus* infections (**Figure 1**). Of the 95 enrolled patients, 93 presented with skin and soft-tissue infections, and one each with bacteremia and a urinary tract infection respectively.

### MRSA risk factor questionnaire

A structured questionnaire was administered to index participants to collect demographic information and assess risk factors for MRSA, including individual, health-related or community exposures. Potentially sensitive information was obtained using audio computer-assisted self-interviewing (ACASI). Data were collected at both the individual and household level. In addition to obtaining index case histories of past infections, clinical information on recurrent infections was obtained from the index case medical records.

### Microbiological sample collection and molecular studies

Anterior nares cultures were collected with sterile pre-moistened swabs (Becton Dickinson) from index participants and consenting household members, excluding children <1 year old. The average number of nasal swabs collected from case households was similar to those from control households (mean 3.3 versus 3.2, P = .66).

In each house a standardized list of environmental items were sampled with pre-moistened swabs: three door knobs (entrance, bathroom, bedroom), TV or game remote, living room light switch, two toys, couch or bed, computer or radio, house phone or index cellular phone, bathroom sink, and kitchen appliance handle. The average number of swabs analyzed for case and control households respectively was comparable (mean 7.9 for both, p = .95). Culture swabs were incubated overnight at 37°C in high-salt 6.5% broth, and plated onto Mannitol Salt Agar (Becton Dickinson), for 48 h at 35°C. Positive, mannitol-fermenting yellow colonies were isolated onto 5% Sheep Blood Agar plates (Becton Dickinson) and single colonies were selected for further analysis. *S. aureus* was identified by coagulase and Protein A detection kit (Murex StaphAurex) [22]. All clinical MRSA isolates of index case subjects were retrieved from the clinical microbiology laboratory.

*S. aureus* isolates were genotyped by *spa*-sequencing, analyzed by Ridom-staphsoftware [23], [24], and subsequently clustered into *spa*-Clonal-Complexes (*spa*-CC) if the cost distances were <4 using the integrated BURP (Based Upon Repeat Patterns) algorithm. *Spa*-types with <5 repeats were excluded from cluster analysis and these *spa*-types were treated as singletons. Representative samples from individual households were subjected to pulsed-field gel analysis (PFGE) and analyzed using BioNumerics-software-v.4.00 (Applied-Maths) to verify close relatedness of strains [10]. Methicillin-resistance was assessed by presence and type of Staphylococcal Chromosomal Cassette (SCC)*mec* using multiplex PCR [25], [26]. The presence of the arginine-catabolic mobile element (ACME) was determined by PCR [27].

### Statistical Analysis

Data were analyzed using SAS 9.1 software (SAS Institute Inc., Cary, North Carolina). Comparison of *S. aureus* colonization between case and control groups was carried out on three different levels: (1) any household surface colonized, (2) nasal colonization index participant only, (3) nasal colonization of non-index household members, and calculated as the ratio of positive swabs to swabs collected per household. McNemar's test was used to compare case and control groups on dichotomous variables and the Signed Rank test for continuous variables obtained from the questionnaire. Conditional logistic regression was used to examine risk factors for MRSA infection and to calculate odds ratios with 95% confidence intervals. A subgroup analysis was conducted among cases to assess the concordance of infectious strains to corresponding colonizing nasal and environmental isolates. Chi-square tests were used to assess whether colonization levels differed depending on occurrence of repeated infections. All statistical tests were 2-sided tests. p<.05 was considered statistically significant.

## Results

This case control study included 95 patients with CA-MRSA infections and their 95 age-matched controls from all age groups with a median age of 30 (range 2–76) for cases and 29 (range 2–75) for controls. There were no significant differences between case patients and control participants regarding their demographic characteristics and healthcare exposures (**Table 1**), or their household and community exposures (**Table 2**). Significantly more cases (15%) than controls (7%) reported diabetes mellitus as an underlying health condition (*P* = .04). Case indexes (36%) more frequently than control indexes (1%) reported a skin infection in the previous 6 months other than the one that led to recruitment into the study. Household members living with case subjects (26%) also reported more skin infections over the past 6 months than those sharing a household with control participants (7%). Indexes from both groups reported low levels of drug use and recent incarceration, including among other household members (<1% each).

**Table 1:** Study Population Characteristics, by Case-Control Status.

		CasesN = 95 (%)		ControlsN = 95 (%)		*P*-value^a^
**Demographics**						
Gender	Female	59	(62)	69	(73)	.09
	Male	36	(38)	26	(27)	
Age category (years)	<5	8	(8)	7	(7)	.73
	5–17	15	(16)	19	(20)	
	18–24	14	(15)	12	(13)	
	25–44	30	(32)	28	(30)	
	>45	28	(29)	29	(30)	
Race/ethnicity	Latino	71	(75)	74	(78)	.62
	Other	24	(25)	21	(22)	
Household income	< $21,000	72	(76)	71	(75)	.87
	> $21,000	23	(24)	24	(25)	
Education	<High School graduation	46	(52)	58	(61)	.14
	>High School graduation	42	(48)	37	(39)	
**Comorbidities and health-related exposures**						
Diabetes mellitus, index	Yes	15	(16)	7	(7)	**.045**
	No	80	(84)	88	(93)	
HIV, index	Yes	3	(4)	4	(5)	.71
	No	76	(96)	69	(95)	
Antibiotic use other household members	Yes	33	(35)	35	(38)	.65
	No	61	(65)	56	(62)	
SSTI other household members	Yes	22	(26)	6	(7)	**.004**
	No	62	(74)	80	(93)	
Antibiotic use, index^b^	Yes	93	(98)	42	(44)	**<.0001**
	No	2	(2)	53	(56)	
Recurrent infections, index, past 6 months^c^	Yes	36	(36)	1	(1)	**<.0001**
	No	58	(64)	93	(99)	

a McNemar's Test was used to compare dichotomous variables and the Signed Rank test used for continuous variables.

b Includes treatment received for infection leading to recruitment into the study.

c Among cases, excludes infection that led to recruitment into this study.

**Table 2:** *S. aureus* Risk Factors, by Case-Control Status.

		CasesN = 95 (%)		ControlsN = 95 (%)		*P*-value^a^
**Household and hygiene exposures**						
Towel sharing	Yes	22	(23)	15	(16)	.19
	No	73	(77)	80	(84)	
Razor sharing	Yes	10	(10)	9	(9)	.80
	No	85	(90)	86	(91)	
Pet in house	Yes	27	(28)	29	(31)	.76
	No	68	(72)	66	(69)	
Single households	Single member	11	(12)	7	(7)	.21
	Multiple members	84	(88)	88	(93)	
Household members	Mean (SD)	4.1	(2.0)	4.1	(2.0)	.82
	Median (Min, Max)	4.0	(1, 10)	4.0	(1, 10)	
Rooms per house	Mean (SD)	3.3	(1.2)	3.0	(1.3)	.47
	Median (Min, Max)	3.0	(1, 8)	3.0	(1, 8)	
HH* members per room	Mean (SD)	1.4	(1.0)	1.5	(0.9)	.44
	Median (Min, Max)	1.2	(0.3, 7)	1.3	(0.25, 6)	
Overnight visitors	Yes	42	(44)	34	(36)	.24
	No	53	(56)	61	(64)	
Number of visitors	Mean (SD)	0.89	(1.41)	0.62	(1.02)	.26
	Median (Min, Max)	0	(0, 7)	0	(0, 5)	
**Community exposures, index**						
Travel past 6 months	Yes	18	(19)	23	(24)	.37
	No	77	(81)	72	(76)	
Daycare attendance	Yes	3	(3)	6	(6)	.08
	No	92	(97)	89	(94)	
Sports participation	Yes	25	(26)	23	(24)	.72
	No	75	(74)	72	(76)	
Recent tattoo	Yes	5	(5)	1	(1)	.10
	No	90	(95)	94	(99)	
Recent ear piercing	Yes	4	(4)	3	(3)	.71
	No	91	(96)	92	(97)	

a McNemar's Test was used to compare dichotomous variables and the Signed Rank test for continuous variables.

*HH  =  household

### Exposure to environmental *S. aureus* contamination and nasal *S. aureus* colonization

**Figure 2:**
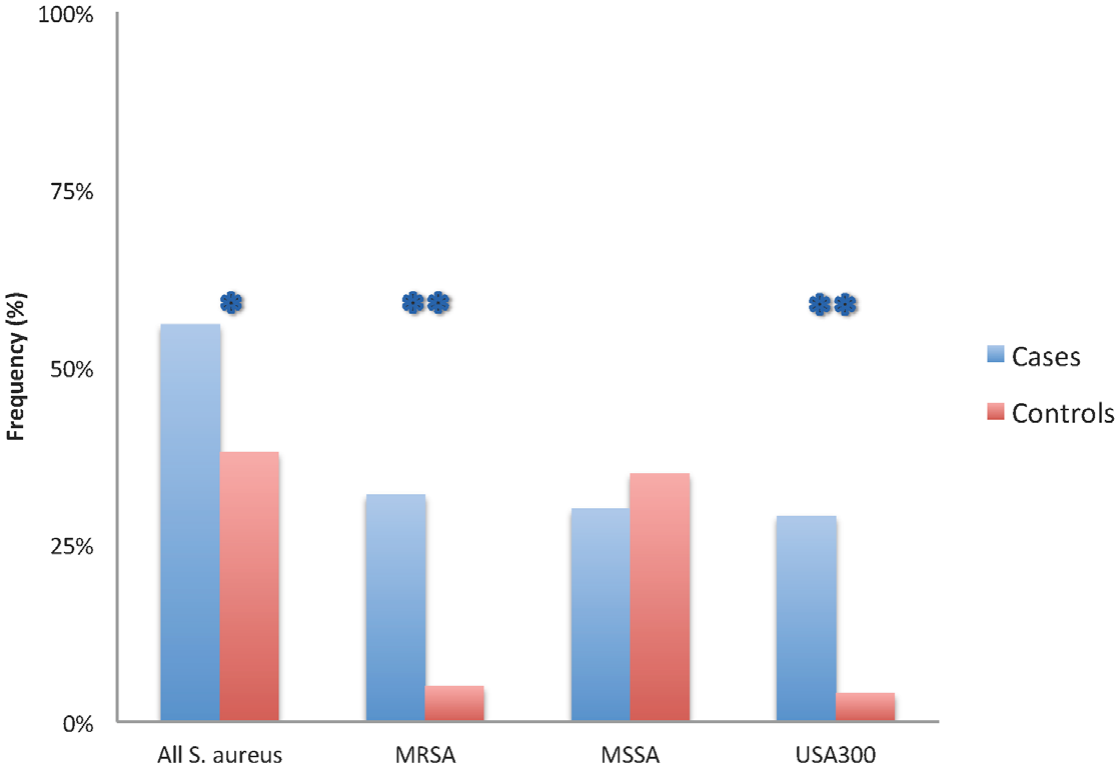
Comparison of frequency of contamination of household surfaces with *S. aureus* overall, MRSA, MSSA or USA300 by case (blue) or control (red) group status. * denotes p < 0.05 and ** p < 0.01.

We detected environmental contamination with *S. aureus* in more than half of the case households (53/95, 56%) as compared to 36/95(38%) control households (*P* = .02; **Table 3**). This difference was mainly due to MRSA being recovered from environmental surfaces in almost one third of case households (32%) compared to only (5%) of control households (*P*<.001), whereas MSSA was detected at about equal frequencies in houses from both groups (30% versus 35%; **Figure 2**). MSSA and MRSA were detectable at any of the standardized items tested, but were predominantly recovered from doorknobs and couches in both, case and control households.

**Table 3:** Colonization of Environment and Nares Among Cases and Controls.

		CasesN = 95 (%)		ControlsN = 95 (%)		OR [95% CI]	*P-*value
**Household environment colonizedon at least one surface**							
*S. aureus*	Yes	53	(56)	36	(38)	2.1 [1.1–4.1]	**.02**
	No	42	(44)	59	(62)		
MSSA	Yes	29	(30)	33	(35)	0.9 [0.5–1.6]	.60
	No	66	(70)	62	(65)		
MRSA	Yes	30	(32)	5	(5)	6.8 [2.4–19.4]	**<.001**
	No	65	(68)	90	(95)		
**Nasal colonization, index**							
*S. aureus*	Yes	27	(28)	29	(30)	0.9 [0.5–1.7]	.75
	No	68	(72)	66	(70)		
MSSA	Yes	7	(7)	27	(28)	0.2 [0.1–0.5]	**<.001**
	No	88	(93)	68	(72)		
MRSA	Yes	20	(21)	2	(2)	12.4 [2.8–54.7]	**<.001**
	No	75	(79)	93	(98)		
**Proportion of nasal colonization, other household members** a							
*S. aureus*	Mean (SD)	0.91	(1.0)	0.68	(1.2)	1.2 [0.9–1.6]	.15
MSSA	Mean (SD)	0.60	(.86)	0.61	(1.14)	1.0 [0.7–1.3]	.98
MRSA	Mean (SD)	0.31	(0.58)	0.07	(0.39)	2.7 [1.3–5.6]	**<.005**

Abbreviations: OR, odds ratio; CI, confidence interval.

a Proportion of nasally colonized household members per house (indexes excluded).

We did not detect any difference in frequency of nasal colonization with *S. aureus* between case (28%) and control indexes (31%; **Table 3**). However, case indexes were more likely to be nasally colonized with MRSA (21%), compared with only 2% of control indexes. Conversely, control indexes had a higher prevalence of nasal MSSA colonization than cases. We then compared case and control groups for the frequency of nasal colonization among other household members. Persons living in case households were found to be more often nasally colonized with MRSA than were people residing in control households, whereas they did not differ in their frequency of nasal MSSA or overall *S. aureus* colonization (**Table 3**).

### Molecular characterization of *S. aureus* isolates

**Figure 3:**
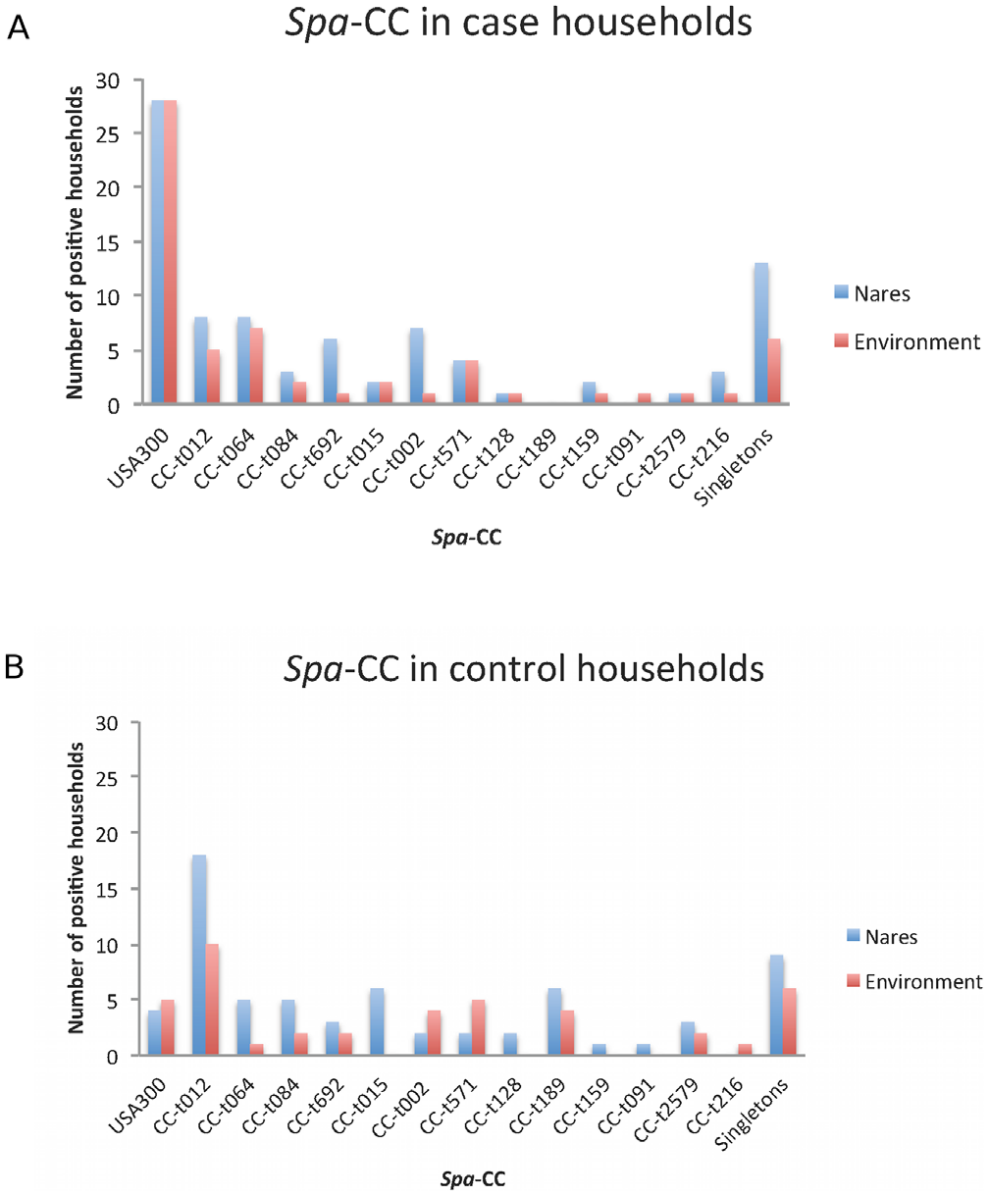
Distribution of *S. aureus spa*-CC in environmental (panel A) and nasal (panel B) colonization between case (blue) and control (red) households. Genotyping of all isolated nasal and environmental *S. aureus* strains yielded 83 different *spa*-types that were clustered into *spa*-CC by BURP analysis. *Spa*-types with < 5 repeats were excluded from clustering and are summarized as singletons. Individual households may be represented more than once, which reflects that they harbored multiple strains.

We detected 83 different *spa*-types among the 223 *S. aureus* isolates from case households and 169 *S. aureus* strains from control households. These clustered into 13 *spa*-CC (**Figure 3**). USA300 was the most frequently cultured strain from the nares of at least one person and from at least one environmental object (28 households each) in case households. In contrast, USA300 was rarely retrieved from control households (4 nasal and 5 environmentally positive households), where the clone CC-t012/ST30 predominated (18 nasal and 10 environmental positive households). In contrast, this clone was less frequently recovered from case households (8 nasal and 5 environmentally positive households).

USA300-ST8 strains (n = 77, 81%), including *spa*-t008 (n = 73) and *spa*-t024, t211 (n = 4), [28], were also the source of most clinical infections. The remaining 18 (18.9%) infectious isolates belonged to 10 different *spa*-types that clustered into 3 separate *spa*-CC. In about one-third of the case households, the concordant clinical infectious isolate was recovered from an environmental surface (31/95, 33%), but was less frequently cultured from the nares of either the index (19/95, 20%) or other household members (18/68, 26%). Of the 77 households with index infections caused by USA300, 28 (36%) were contaminated environmentally with USA300, compared to 4/18 (22%) of infections caused by other MRSA strains (*P* = .25). We observed an inverse trend for nasal colonization, where out of the 77 patients with USA300 infections only 13 (17%) were also nasally colonized with USA300, compared to 6/18 (33%) of those infected with other MRSA strains, though this difference did not reach statistical significance (*P* = 0.19). ACME, a potential fitness-enhancing factor almost exclusively restricted to USA300 [6], [29], was present in 67 of the clinical isolates (71%). All but one (*spa*-t216/ST-59) of these samples were USA300, whereas ACME was lacking in 11/77 USA300 isolates. The presence of ACME in clinical infectious isolates did not affect the ability of strains to contaminate environmental surfaces (38/54, 71%) as compared to households with an infection caused by a strain lacking this element (29/41, 71%, *P* = .58).

### Subgroup analysis recurrent infections and strain-type

**Figure 4:**
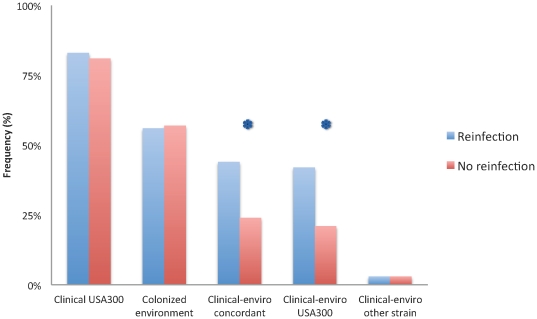
Comparison of *S. aureus* infectious isolate and environmental contamination between cases with reinfection (blue) or without recurrent infections (red). Shown are the frequencies of USA300 as the clinical infectious isolate, the colonized environment, the houses with clinical and environmental strain concordance (Clinical-enviro concordant), the clinical and environmental concordant strain being USA300 (Clinical-enviro USA300), or the clinical and environmental strain being a clone other than USA300 (Clinical-enviro other strain). * denotes p < 0.05.

Ninety-four of the 95 index cases provided information on antecedent infection within the six months preceding enrolment. Of these, 36 (38%) subjects either reported or had an infection documented in their clinical record other than the infection that led to recruitment into the study. There was no difference in the type of clinical isolate or the overall proportion of positive environmental households between cases with or without reinfections (**Table 4**). However, among households with recurrent infections, almost half (44%) had at least one environmental item contaminated with the same strain that caused their clinical infection. This differed significantly from households in which the individual did not have a recurrent infection. Here, only one quarter of the households (24%) were environmentally colonized with the same strain (*P* = .04; **Figure 4**). Moreover, this difference in environmental contamination was mainly accounted for by USA300 strains (*P* = .02). In contrast, no differences in nasal colonization of index cases were observed between reinfection and non-reinfection groups (**Table 4**). No other risk factors identified in the case-control analysis, such as diabetes mellitus of the index or infections in other household members, were detected between the groups with and without recurring infections (data not shown).

**Table 4:** *S. aureus* Colonization Among 94 Case Households by Presence of Antecedent Infection*.

	Reinfection N = 36		No reinfection N = 58			
	N	(%)	N	(%)	OR (95% CI)	*P*-value^a^
**Environmental colonization**						
Colonized environment	20	(56)	33	(57)	1.0 [0.4–2.2]	.89
Clinical isolate/environment concordant	16	(44)	14	(24)	2.5 [1.0–6.1]	**.04**
Clinical isolate (USA300)	30	(83)	47	(81)	1.2 [0.4–3.5]	.78
Clinical isolate/environment concordant (USA300)	15	(42)	12	(21)	2.7 [1.1–6.9]	**.03**
Clinical isolate (other strain)	6	(17)	11	(19)	0.9 [0.3–2.6]	.78
Clinical isolate/environment concordant (other strain)	1	(3)	3	(3)	0.5 [0.1–5.2]	1.0
**Index nasal colonization**						
Colonized nares, index	13	(36)	13	(22)	2.0 [0.8–4.9]	.15
Clinical isolate/nasal concordant	9	(25)	8	(14)	2.1 [0.7–6.0]	.17
Clinical isolate/nasal concordant (USA300)	6	(17)	6	(10)	1.7 [0.5–5.9]	.38
Clinical isolate/nasal concordant (other strain)	3	(8)	3	(4)	2.6 [0.4–16.0]	.32

*Data missing for one case.

a Using chi-square analysis.

## Discussion

In this community-based study we have established a direct relationship between *S. aureus* environmental contamination in households and CA-MRSA infections. First, we detected *S. aureus* and in particular MRSA more frequently on fomites in case than in control households. Second, molecular characterization identified the infectious MRSA isolate on fomites in about one-third of case households, suggesting that this represents a potential infectious reservoir for community-dwelling patients with MRSA. Third, subjects with MRSA infections were more likely to have had antecedent infections than controls. Among these patients with recurrent infections, environmental contamination with the epidemic strain USA300, but not nasal colonization, was observed more frequently. Furthermore, in our diverse study population representing all age groups from the community, we found that case patients more frequently were diabetic and reported skin infections among their household members.

Our observation that *S. aureus* can be detected on fomites in control households also suggests that the ability to survive outside the body is not unique to MRSA or to infectious isolates. Enhanced survival in the environment is therefore likely a phenomenon that is shared between USA300 and other successful strains. Recent survival studies have shown that certain *S. aureus* strains can persist between seven days and seven months on environmental surfaces [30], [31], [32]. Survival may be limited when no organic material such as soil or pus, protects bacteria from desiccation [33]. We note that an *in vitro* study comparing survival of outbreak versus sporadic MRSA isolates demonstrated slowly decreasing viability for all isolates. However, epidemic strains III-29 and III-215, which had caused an outbreak in a local hospital, survived the longest [30].

The observation of an inverse trend between relatively lower index nasal colonization and higher environmental contamination among cases with USA300 infections compared to other *S. aureus* clones, further supports the theory that strain-specific characteristics promote adaptation to body niches and improve survival on inert surfaces. This phenomenon did not appear to be mediated by ACME, which is almost exclusively detected in USA300 and as been implicated as a factor that enhances fitness [6], [29], [34]. Alternatively, differences in environmental contamination between cases and controls could also be explained by differences in colonization of non-nasal body sites (*i.e.* pharyngeal, inguinal, perianal or vaginal [35], [36], [37]) of infected patients and their household members [10], [38], which was not evaluated with the current study design. There is growing evidence that CA-MRSA in particular may have a higher propensity to colonize groin and genital areas and serve as a source for infections, in particular with USA300 [37]. This enhanced capacity to colonize non-nasal body sites may in turn enable increased transmission of *S. aureus* between individuals [39] as well as shedding into the environment. In this scenario increased environmental contamination with USA300 could be an indicator for overall higher burden of non-nasal carriage rather than improved survival on inert surfaces.

The degree of environmental contamination may also be a reflection of hygiene practices. However, we were unable to identify potential confounders of hygiene with our study design, as environmental *S. aureus* colonization was not related to sharing of items [10], [38], using bleach (not shown), household size or crowding.

Several limitations to our study need to be considered. First, this is a retrospective observational study that only assessed *S. aureus* carriage after infection was established and treated. This study was therefore not able to assess the directionality of *S. aureus* contamination and infection of case indexes. However, the observation that patients with contaminated home surfaces also reported recurrent infections further suggests an important role of the environment as a reservoir for *S. aureus*. Second, these results are representative of a single, predominantly Hispanic community in Northern Manhattan and may have limited generalizability. Third, the overwhelming presence of USA300 in the clinical samples precluded more detailed comparisons of other CA-MRSA and their environmental colonization properties in the subgroup analysis of cases. In addition, analysis of environmental colonization on the household level can also be strongly influenced by the number of sampled surfaces. In a recent pilot study, 34 of 35 households were positive for *S. aureus* when 32 surfaces were tested per home [20], [21]. In comparison, we selected the most frequently colonized sites from this pilot and cultured a mean of nine surfaces per household. The proportion of positive environmental samples per household may be a more meaningful measure than the presence of *S. aureus* only, when assessing the potential impact of contamination on infections.

Interestingly, antecedent infections were more frequently reported by subjects from households with currently contaminated surfaces. Again, this was predominantly due to the presence of USA300 in the environment. There are several possible explanations how environmental contamination can contribute to recurrent infections. First, environmental contamination may serve as a source for recolonization of subjects, whose infection and carrier status had been successfully eliminated by treatment with antibiotics. Second, environmental contamination may be a surrogate marker of either colonization of multiple body sites other than the nares, or of colonization of multiple household members. There has been mounting evidence that the inguinal region or the throat are more frequently colonized with CA-SA and that only assessing nasal colonization may underestimate the true burden of *S. aureus* carriage in individuals in the community [17], [35], [36]. In addition, we observed that the identical clinical isolate was recovered from about a quarter of case household members. Spread of *S. aureus* from person to surface to person appears to be a plausible mechanism of transmission among household members. Third, our results also suggest that the presence of USA300 in the environment was associated with recurrence of infections. This further indicates that USA300 may have unique genotypic and phenotypic features that allow for better survival in environmental niches, which in turn may be a major driving force for the recent dramatic increase in skin infections caused by CA-MRSA.

The ongoing CA-MRSA epidemic urgently requires new intervention and prevention strategies, as conventional management with antibiotics or drainage of abscesses appears unable to curb the spread of these infections. Our results suggest that contamination of household surfaces may play a major role in CA-MRSA transmission, particularly with the epidemic strain USA300. These observations may have major clinical implications in managing CA-MRSA infected patients and warrant further validation in a prospective study.
